# Two-Sample Network Mendelian Randomization and Single-Cell Analysis Reveal the Causal Associations and Underlying Mechanisms Between Antihypertensive Drugs and Kidney Cancer

**DOI:** 10.7150/jca.110850

**Published:** 2025-06-12

**Authors:** Ruiyi Deng, Mingrui Zou, Jianhui Qiu, Jiaheng Shang, Chaojian Yu, Peidong Tian, Yizhou Wang, Lin Cai, Jingcheng Zhou, Kan Gong

**Affiliations:** 1 Department of Urology, Peking University First Hospital, Beijing, China.; 2 Institute of Urology, Peking University, Beijing, China.; 3 National Urological Cancer Center, Beijing, China.; 4 Department of Central Laboratory, Peking University First Hospital, Beijing, China.

**Keywords:** antihypertensive drugs, drug target, kidney cancer, Mendelian randomization, single-cell analysis

## Abstract

**Background**: Antihypertensive drugs represent the most widely used drugs worldwide. However, the association between antihypertensive drugs and the risk of kidney cancer remains unclear. This study innovatively integrates multi-omics and causal inference approaches to investigate the long-term effects and potential mechanisms of 12 antihypertensive drug classes on kidney cancer risk.

**Methods:** In this study, novel approaches including two-sample mendelian randomization (MR), summary-data-based mendelian randomization (SMR), two-step network MR, and single-cell transcriptomic analysis were employed. Single nucleotide polymorphisms (SNPs) were obtained from genome-wide association studies (GWASs) to proxy exposures and outcomes. The cis-expression quantitative trait loci (cis-eQTL) as the proxies of exposure were also obtained. MR estimates were generated using the inverse-variance weighted method or Wald ratio method. Sensitivity analyses were undertaken to interrogate the robustness of the main findings. Two-step network MR and single-cell analysis were specifically designed to dissect pathway-level mediation and expression patterns of identified targets.

**Results**: In the main analysis, genetically proxied calcium-channel blockers (odds ratio [OR]: 0.95, 95% confidence interval [CI]: 0.91-0.99, p=0.021) and vasodilator antihypertensives (OR: 0.86, 95% CI: 0.76-0.97, p=0.018) were suggestively associated with decreased risk of kidney cancer, whereas genetically proxied angiotensin-converting enzyme inhibitors (OR: 1.13, 95% CI: 1.00-1.27, p=0.043) was suggestively associated with increased risk of kidney cancer. Genetically proxied antiadrenergic agents (OR=0.94, 95% CI: 0.90-0.99, p=0.021) and centrally acting antihypertensives (OR=0.93, 95% CI: 0.88-0.98, p=0.010) were suggestively associated with a decreased risk of clear cell renal cell carcinoma. SMR analysis revealed that these suggestively significant associations might be driven by *CACNA1C*, *CALM1*, *ACE*, and *LTA4H*. Upon two-step network MR analyses, 10 pathways with directional consistency were identified, and the mediation proportion ranged from 3.22% to 7.12%. The influence of antihypertensive drugs on kidney cancer risk might be associated with their regulation of levels of blood cells and lipids. Single-cell analysis further revealed the expression patterns of the four identified targets in peripheral blood and tumor infiltrating immune cells.

**Conclusion:** This study pioneers the integration of causal inference and single-cell omics to demonstrate that antihypertensive drugs modulate kidney cancer risk through target-specific mechanisms involving blood cell and lipid pathways. Our findings provide actionable targets (*CACNA1C*, *CALM1*, *ACE*, and *LTA4H*) for drug repurposing trials and underscore the clinical importance of personalized antihypertensive therapy in cancer prevention.

## 1. Introduction

The incidence rate of kidney cancer increased during the most recent years, with more than 400,000 new diagnoses every year worldwide 1. Kidney cancer contains a heterogeneous group of cancers. Renal cell carcinoma (RCC) constitutes over 90% of all solid kidney cancer, predominantly including clear cell RCC (ccRCC, 70%), papillary RCC (pRCC, 10-15%), and chromophobe RCC (chRCC, 5%) 2,3. The risk factors for kidney cancer can be grouped as either non-modifiable factors or modifiable factors, with previously established modifiable risk factors for the development of kidney cancer including excess body weight, smoking, and hypertension 4-6. Other risk factors such as the use of antihypertensive drugs, the type of diet, and physical activity are still not well-studied. The role of these factors in kidney cancer development and prognosis still needs to be investigated 7.

Hypertension affects more than 1 billion people worldwide 8, and is an important risk factor for various health conditions 9. Antihypertensive drugs, used by 30-70% of patients worldwide, represent one of the most prescribed drug classes 10. As lifelong treatments, even minor adverse effects could have significant population-level consequences 11. While numerous adverse effects of antihypertensive drugs have been intensively studied, their possible oncogenic effects have gained the attention of the scientific community for many years. Though the positive association between hypertension and kidney cancer has been reported in several studies 12,13, the impact of antihypertensive drugs on kidney cancer development remains inconclusive. A recent meta-analysis found a 2% higher incidence of kidney cancer with the use of antihypertensive drugs, particularly angiotensin-converting enzyme inhibitors (ACEIs) and angiotensin II receptor blockers (ARBs) 14, contrasting with studies suggesting that antihypertensive drugs may exert protective effects through antiangiogenic mechanisms and calcium regulation 15,16. Furthermore, some studies reported that no significant association was found between antihypertensive drugs and the risk of kidney cancer 17-19. These inconsistencies may originate from the multigenic and heterogeneous trait of kidney cancer, and confounding factors in observational studies, including unmeasured variables affecting pharmaco-epidemiological analyses 20,21. Therefore, the evidence level of previous studies is relatively low, which makes it unable for us to accurately establish causal relationships between antihypertensive drugs and kidney cancer. The criterion standard approach for determining the causal effect could be randomized clinical trials (RCTs) 22. However, given that most hypertensive patients receiving lifelong antihypertensive treatment, and developing kidney cancer may take decades, RCTs are expensive and may not be feasible. This may explain a current lack of large-scale, high-quality RCTs assessing the effect of antihypertensive deugs on kidney cancer.

In recent years, using human genetics to assess the efficacy and safety profiles of therapeutic targets has become increasingly popular in drug development 23,24. Mendelian randomization (MR), utilizing genetic variants as instruments to perform causal inference between exposure and outcome, has been proposed to predict drug repurposing opportunities and overcome some of the shortcomings in previous studies 25-27. Unlike traditional observational studies that are often confounded by residual factors and reverse causality, MR minimizes these biases by utilizing germline genetic variants that are randomly assorted at meiosis 28. Since these genetic variants are assigned before disease development, MR analysis not only allows for the assessment of long-term modulation of drug targets on cancer risk but also effectively avoids reverse causality 20. As a result, conclusions provided by MR analysis could be comparable in evidence strength to those derived from RCTs, offering a cost-effective, convenient, and reliable alternative for researchers.

In this study, we used naturally occurring variations on genes encoding antihypertensive drug targets as proxies for these targets to investigate the effect of their therapeutic inhibition on the risk of kidney cancer. In addition, given that previous studies have not yet explored the mechanisms by which antihypertensive drugs influence the risk of kidney cancer, we further employed Summer-data-based mendelian randomization (SMR), two-step network MR and single-cell analysis to delve deeper into this area. Through these approaches, we identified potential mediators and target genes. Our study would provide evidences for the etiological research of kidney cancer. A greater understanding of antihypertensive drugs and their effect on kidney cancer development may shed light on potentially relevant biological mechanisms for kidney cancer.

## 2. Materials and Methods

This study was reported according to the statement for strengthening the reporting of observational studies in epidemiology using Mendelian randomization (STROBE-MR) guideline 29. All cited genome-wide association studies (GWASs) included in our analyses had the relevant institutional review board approval, following the Declaration of Helsinki. All participants had provided informed consent.

### 2.1. Study design

**Figure 1:**
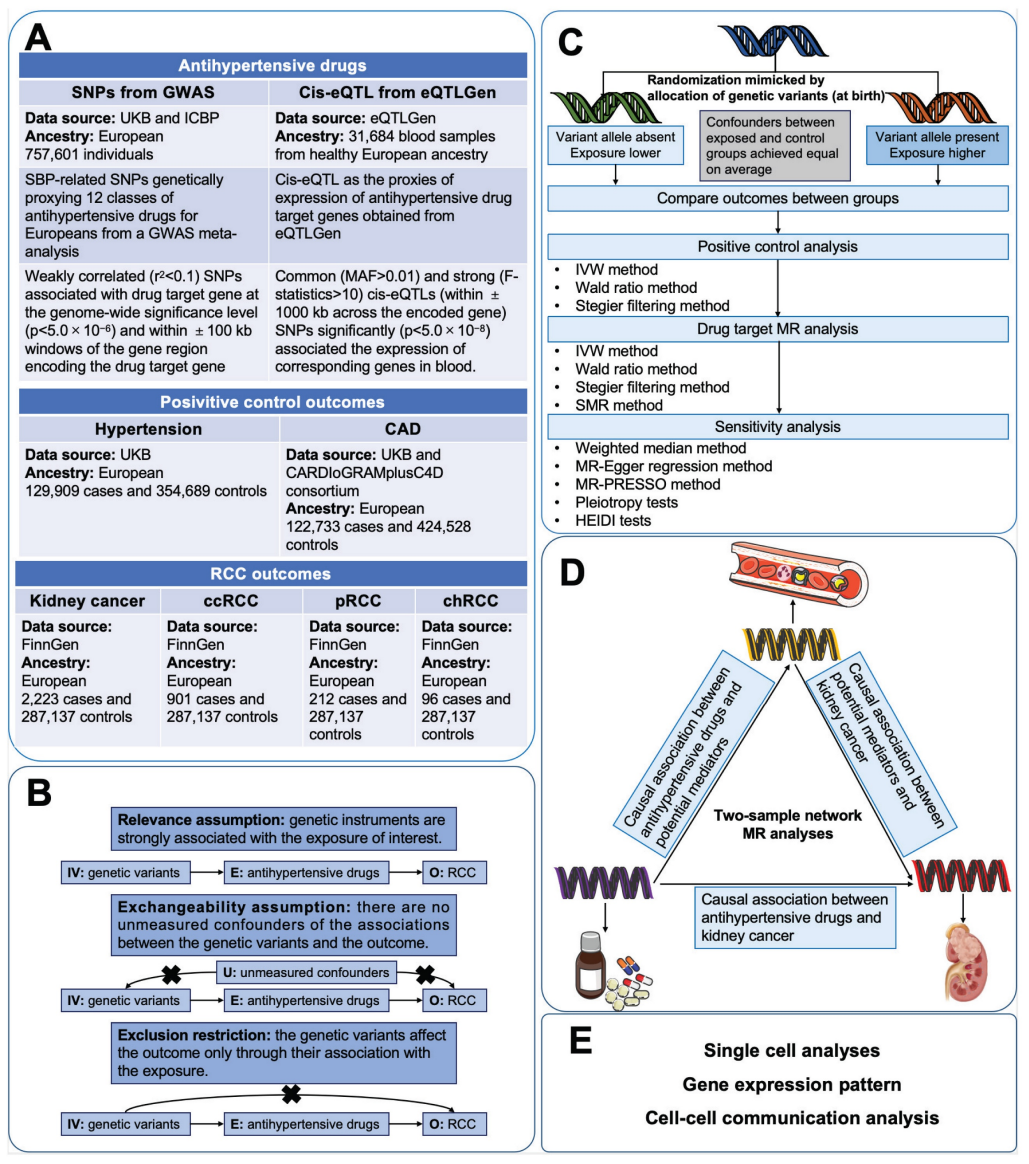
Study design. Abbreviations: CAD, coronary artery disease; ccRCC, clear cell renal cell carcinoma; chRCC, chromophobe renal cell carcinoma; CI, confidence interval; GWASs, genome-wide association studies; IV, instrumental variable; IVW, inverse-variance weighted; MR, mendelian randomization; pRCC, papillary renal cell carcinoma; SNP, single nucleotide polymorphisms; SMR, Summer-data-based Mendelian Randomization.

** Figure 1** illustrated the study design of this investigation. Firstly, we conducted two-sample MR analyses to explore the causal associations between genetically proxies for antihypertensive drugs and kidney cancer. Secondly, SMR analyses were employed to identify possible causal genes driving these associations. In the subsequent phase, we conducted two-step MR analyses to assess the mediation effects of blood cells, blood lipids and anthropometric measurements in the association between antihypertensive drugs and kidney cancer. Finally, single-cell analysis was conducted to reveal the expression patterns of the identified antihypertensive drug targets genes causally associated with kidney cancer.

### 2.2. Data sources and study populations

Antihypertensive drugs were categorized according to the Anatomical Therapeutic Chemical classification system. 12 classes of antihypertensive drugs were incorporated in this study, including antiadrenergic agents (including ganglion-blocking and peripherally acting), alpha-adrenoceptor blockers, ARBs, ACEIs, beta-adrenoceptor blockers (BBs), CCBs, centrally acting antihypertensives, loop diuretics, potassium-sparing diuretics (PSDs) and aldosterone antagonists, renin inhibitors, thiazides and related diuretics, and vasodilator antihypertensives. The genes encoding the targets of the 12 classes of antihypertensive drugs were identified from the DrugBank database (**Table S1**) 30. The gene information of drug targets was obtained from the National Cancer for Biotechnology Information database. In primary analyses, genetic instrumental variables (IVs) of systolic blood pressure (SBP) for Europeans were obtained from a GWAS meta-analysis of the International Consortium of Blood Pressure including 757,601 participants 31,32. The cis-expression quantitative trait loci (cis-eQTL) as the proxies of exposure were obtained from eQTLGen (https://www.eqtlgen.org/) and a previously published study 33.

The genetic IVs of risk of hypertension (129,909 cases; 354,689 controls of European ancestry) and coronary artery disease (CAD) (122,733 cases; 424,528 controls of European ancestry) were chosen for accessing the validity of genetic instruments of SBP 34,35. For kidney cancer outcomes, the latest summary genetic association estimates for overall kidney cancer risk in up to 2,223 cases and 287,137 controls were obtained from the FinnGen study 36. Summary genetic association data of clear cell RCC (ccRCC), papillary RCC (pRCC), and chromophobe RCC (chRCC) was retrieved from the FinnGen study 36.

As previous studies have demonstrated that antihypertensive drugs exerted specific regulatory effects on the levels of blood lipids and blood cells, and may also affect some anthropometric parameters (such as BMI) 37-41. We aimed to explore whether these variables played mediating roles in the associations between antihypertensive drugs and kidney cancer. GWAS data of potential mediators (Blood cells, blood lipids and anthropometric measurements) all came from IEU open GWAS (https://gwas.mrcieu.ac.uk). Details of data sources were shown in **Table S2**.

### 2.3. Genetic IVs construction

To proxy antihypertensive drugs, SNPs associated with the drug target gene at the genome-wide significance level (*p*< 5.0×10^-6^) and within ±100 kb windows of the gene region encoding the drug target gene were obtained. The genetic variants in a relevant coding region are known as cis-variants, which could be taken as a measure of pharmacological perturbation of the relevant drug target 42,43. To maximize the instrument strength, SNPs used as IVs were only permitted to be in low weak linkage disequilibrium (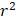
<0.1) with each other, so as to increase the proportion of variance in each class of antihypertensive drugs explained by the IVs. To obtain genetic IVs of potential mediators, we chose genome-wide significant (*p*<5×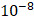) SNPs with low linkage disequilibrium (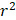
<0.001). Palindromic SNPs and SNPs containing missing data were eliminated.

The MR analysis is based on three core assumptions. The first assumption is that the genetic IVs should be strongly associated with the exposure of interest (At the genome-wide significance level). To avoid weak instrumental bias, F-statistics were calculated for each SNP of genetically proxied antihypertensive drugs. SNPs with F-statistics less than 10 would be excluded 44. The second assumption is that the IVs are not affected by any confounding factors related to either the exposure or the outcome. The third assumption is that the genetic variants affect the outcome only through their association with the exposure 23,45.

When an SNP only presents in the exposure GWAS but not in the outcome GWAS, we would find a proxy SNP in high linkage disequilibrium (r^2^>0.8) through LDlink (https://ldlink.nci.nih.gov/). Before MR analysis, SNPs proxying exposure were harmonized with the SNPs of genetically proxied outcome. If the effects of the IVs on the exposure and the outcome did not correspond to the same allele on the same DNA strand, we would align the allele in the two datasets and flip its genetic effect size accordingly. The detailed information of IVs used in this study is presented in **Table S3** and **S4**.

### 2.4. Mendelian randomization analysis

Positive control MR analysis serves to justify the genetic IVs of the drug by demonstrating the expected effect on the outcome which has an established causal relationship with the drug of interest 46. The intended indication for antihypertensive drugs is hypertension. Furthermore, the prescription of antihypertensive drugs has been recognized as a crucial therapy for reducing the morbidity of CAD 47,48. Therefore, to validate the SNPs as IVs for the antihypertensive drugs, two-sample MR analyses were conducted to examine the associations between genetically proxied antihypertensive drugs and the risk of hypertension and CAD. In the main analysis, the inverse-variance weighted (IVW) method or Wald ratio method was utilized to investigate the causal effect of genetically proxied therapeutic inhibition for antihypertensive drug targets on the risk of kidney cancer 49. For taxa with more than one genetic IVs, IVW was chosen as the main statistical method. For taxa with only one genetic IV, the estimate for causal association was performed by Wald ratio method 45,50. The meta-analysis could integrate the effects of individual antihypertensive drug target gene instruments into a total weighted effect. The main results were presented as the odds ratio (OR) for outcomes per 1mmHg reduction induced by each class of antihypertensive drugs with 95% confidence intervals (CI). The Cochran's Q test, as well as I^2^ statistics, were performed to evaluate the heterogeneity between SNPs 51. In case of using IVW models, fixed-effect IVW models would be applied if there is no heterogeneity, otherwise, random-effect IVW models would be used. Furthermore, to investigate the effect of genetically proxied antihypertensive drugs on the risk of different histological types of RCC, MR analyses were performed to delve into the effect of genetically proxied therapeutic inhibition for antihypertensive drug targets on the risk of ccRCC, pRCC, and chRCC.

For significant causal associations in MR analysis, we further conducted SMR analysis to investigate the causal association between genetically predicted levels of the targeted genes of these drugs and kidney cancer. Blood eQTLs of corresponding genes from the eQTLGen were used as exposures, and GWAS data of kidney cancer were used as outcomes. Common (minor allele frequency (MAF) > 0.01) and significant (p < 5.0 × 10^-8^) cis-eQTLs were selected, and Summary-data-based MR (SMR) software (version 1.3.1) was used to performed the analysis 52.

We further conducted two-sample MR analyses to evaluate the causal associations between antihypertensive drugs and mediators as well as mediators and kidney cancer. Subsequently, two-sample network MR analyses were applied to assess the potential mediating roles of blood cells, blood lipids and anthropometric parameters. For candidate mediators, we would calculate the indirect effect (mediating effect) by multiplying the estimated effect of exposure on mediator by the estimated effect of mediator on outcome 53. The standard errors (SE) for the indirect effects were calculated using delta method 54. The proportion mediated by mediators were further calculated by dividing the indirect effect by the total effect.

For each MR analysis, stegier filtering method was utilized to provide assurance on the directionality of the association, which could mitigate reverse causality. Reverse causality is considered absent when the direction is “TRUE” and the *p*-value < 0.05 55.

Sensitivity analyses were undertaken to interrogate the robustness of the main findings. Different MR methods with different assumptions of horizontal pleiotropy were used to evaluate the robustness of the IVW analysis results and control for pleiotropy, including weighted median, MR-Egger regression, and MR-pleiotropy residual sum and outlier (MR-PRESSO) 56-58. These robust analysis methods provide causal estimates under weaker assumptions than the IVW method, which is more sensitive in detecting horizontal pleiotropy 45. The weighted median could provide robust estimates for the effect even if half of the included SNPs are pleiotropic 56. The MR-Egger regression provides unbiased estimates of association even when all SNPs are genetically pleiotropic 57. MR-PRESSO is able to assess for the presence of horizontal pleiotropic outliers, and provide a corrected estimate via outliers removal 58. However, affected by the SNPs, these results may not be accurate and consistent. When the results of IVW method and these sensitivity analysis methods were inconsistent, we gave priority to the results of IVW 56. In addition, MR-Egger intercept test was also used for assessing potential pleiotropy 57. Furthermore, we searched on Phenoscanner (http://www.phenoscanner.medschl.cam.ac.uk) to evaluate whether the genetic IVs (or their proxies (r^2^ > 0.8)) were associated with other risk factors for antihypertensive drug targets or kidney cancer at genome-wide significance. For SMR analysis, heterogeneity in dependent instruments (HEIDI) test was employed to evaluate whether the observed causal association was caused by linkage scenario (*p*-value of HEIDI test < 0.05 indicated the presence of linkage scenario). The HEIDI test was performed in the SMR software (version 1.3.1) 52.

### 2.5. Single-cell analysis

To further investigate the expression patterns of identified targets, we obtained scRNA-seq data (GSE121636) from the GEO data base (https://www.ncbi.nlm.nih.gov/) 59. As eQTL data were all from peripheral blood of individuals, we chose samples from peripheral blood. This 10X scRNA-seq data were obtained from 3 samples of peripheral and 3 samples of tumor-infiltrating immune cells in renal cell carcinoma patients.

Seurat objects were generated for the cell-gene count matrix of RCC patients using the R package “Seurat” (V.4.4.0) 60. To ensure data quality, the following criteria were applied during the quality control process: (1) Cells with less than 200 or more than 4,000 feature genes were excluded. (2) Cells with more than 10% mitochondrial genes were also considered low-quality cells and were filtered out. Subsequently, data normalization was conducted to mitigate batch effects. Next, we used the “FindVariableFeatures” function of the package “Seurat” to identify 2,000 highly variable genes for principal component analysis (PCA). To further mitigate the influence of batch effects, package “harmony” (V.0.1.1) was used 61. With the help of the “FindNeighbors” and “FindClusters” functions, cells were clustered and were visualized using the uniform manifold approximation and projection (UMAP) method. Clusters were annotated with the help of “Idents” and “Dimplot” functions. Furthermore, to obtain comprehensive insights into the functions of the core cell subclusters, we employed the package “CellChat” (V.1.6.1) to analyze the intercellular communications between the core cell subclusters and other cell subclusters 62.

### 2.6. Statistical analysis

All statistical tests were two-sided. False discovery rate (FDR) correction was conducted to account for multiple testing and adjust the thresholds of significance level. Strong significant evidence was suggested for corrected *p* value < 0.05, and suggestive significant evidence of corrected *p* value > 0.05 but *p* < 0.05. For SMR analysis, *p* < 0.05 was considered statistically significant, as it served as an additional validation of MR analysis. All analyses were performed with packages named TwoSampleMR (version 0.5.7), and MRPRESSO (version 1.0) in R software (version 4.3.1) and SMR software (version 1.3.1).

## 3. Results

### 3.1. Genetic instruments selection and validation

A total of 103 genes whose encoded protein activity has been experimentally demonstrated to be modified by one or more antihypertensive drugs were identified (**Table S1**). Then 2 to 90 SBP-related SNPs genetically proxying 12 classes of antihypertensive drugs for Europeans were identified (**Table S3**). The F-statistics for all selected SNPs ranged from 20.9 to 627.5.

**Figure 2:**
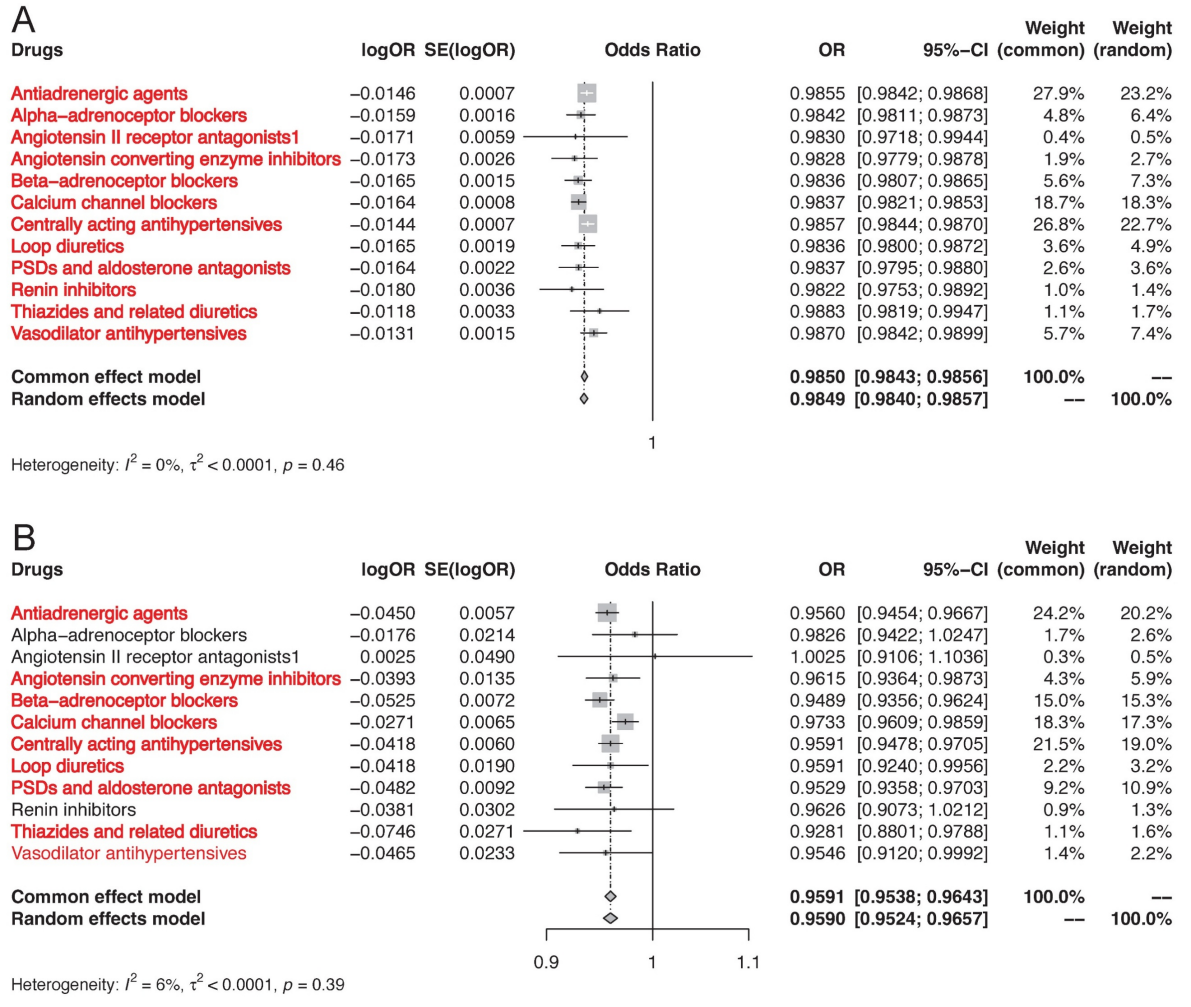
MR association of genetically proxies for antihypertensive drugs with (A) the risk of hypertension, and (B) CAD. Abbreviations: CAD, coronary artery disease; MR, Mendelian randomization. For the specific drug type, if the analysis result was suggestively significant, the name of this specific drug type would be presented in the form of red font. And if the analysis result was strongly significant, the name of this specific drug type would be presented in the form of red bold font.

As shown in **Figure 2**, the results of positive control analysis indicated that genetic variations in the targets of antihypertensive drugs were associated with significant risk reduction in hypertension (**Figure 2A, Table S5**). 9 of the 12 genetically proxied classes of antihypertensive drugs were related to significant risk decrease in CAD except for alpha-adrenoceptor blockers, angiotensin II receptor antagonists, and renin inhibitors (**Figure 2B, Table S6**). The positive control analyses justified the validity of genetic instruments of 12 classes of antihypertensive drugs. These SNPs were included in the primary MR analysis.

### 3.2. MR analysis with antihypertensive drug therapies and kidney cancer risk

**Figure 3:**
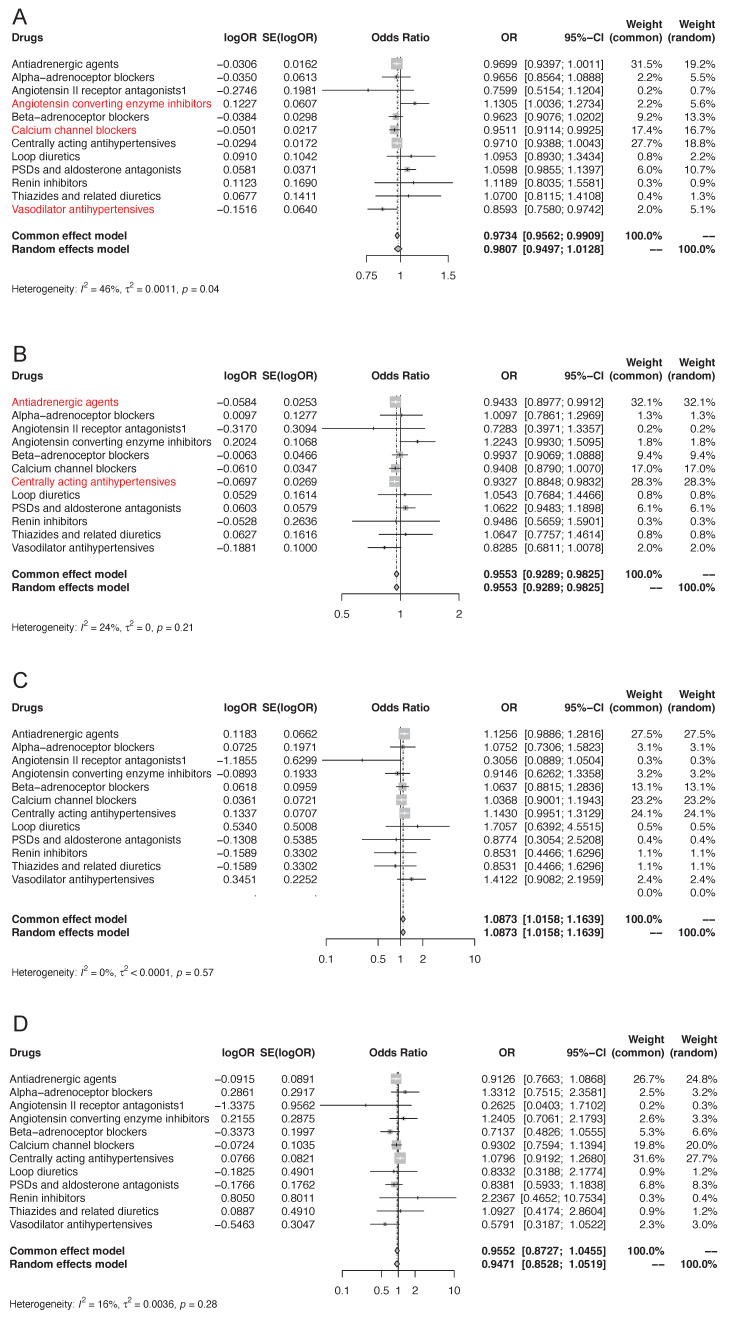
MR association of genetically proxies for antihypertensive drugs with (A) the risk of kidney cancer, (B) ccRCC, (C) pRCC, and (D) chRCC. Abbreviations: ccRCC, clear cell renal cell carcinoma; chRCC, chromophobe renal cell carcinoma; MR, Mendelian randomization; pRCC, papillary renal cell carcinoma. For the specific drug type, if the analysis result was suggestively significant, the name of this specific drug type would be presented in the form of red font. And if the analysis result was strongly significant, the name of this specific drug type would be presented in the form of red bold font.

We first applied drug target MR to investigate the association of genetically proxied antihypertensive drug targets with overall kidney cancer risk (**Figure 3A, Table S7**). IVW-MR analysis demonstrated that there was suggestive evidence of genetically proxied CCBs (odds ratio [OR]: 0.95, 95% confidence interval [CI]: 0.91-0.99, p=0.021) and vasodilator antihypertensives (OR: 0.86, 95% CI: 0.76-0.97, p=0.018) were related to decreased risk of kidney cancer per 1mmHg reduction in SBP. In contrast, there was suggestive evidence that genetically proxied ACEIs were associated with an increased risk of kidney cancer per 1mmHg reduction in SBP (OR: 1.13, 95% CI: 1.00-1.27, p=0.043). There was no evidence of associations between genetic proxies for the other 9 classes of antihypertensive drugs and the risk of kidney cancer (p>0.05). In MR analyses, no heterogeneity was detected for estimating the effect of genetic proxies for all 12 classes of antihypertensive drugs on kidney cancer (p for Cochran Q test >0.05), so fixed-effect IVW models were used. The estimates were similar using weighted median and MR-Egger. No pleiotropy was detected by pleiotropy test and MR-PRESSO global test (p>0.05). All the MR analysis passed Steiger filtering test (**Table S7**). The meta-analysis which integrated the effects of each class of antihypertensive drug target gene instruments into a total weighted random effect showed that there was no significant associations between genetic proxies for overall antihypertensive drug target genes and the risk of kidney cancer (OR=0.98, 95% CI: 0.95-1.01).

To further investigate the influence of antihypertensive drugs on the risk of different histological types of kidney cancer, we performed two-sample MR to study the effect of genetically proxied therapeutic inhibition for antihypertensive drug targets on the risk of ccRCC, pRCC, and chRCC. Initially discovered, genetically proxied antiadrenergic agents (OR=0.94, 95% CI: 0.90-0.99, p=0.021) and centrally acting antihypertensives (OR=0.93, 95% CI: 0.88-0.98, p=0.0096) were suggestively associated with a decreased risk of ccRCC (**Figure 3B, Table S8**). The meta-analysis showed that there was evidence of associations between genetic proxies for overall antihypertensive drug target genes and the risk of ccRCC (OR=0.96, 95% CI: 0.93-0.98). Regarding pRCC and chRCC, there was no evidence of associations between genetic proxies for all 12 classes of antihypertensive drugs (**Figure 3C-3D, Table S9-S10**).

### 3.3. SMR analysis of antihypertensive drug targets and kidney cancer risk

**Figure 4:**
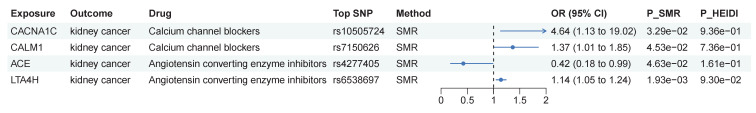
SMR analysis of antihypertensive drug targets and kidney cancer risk. Abbreviations: SMR, summary-data-based Mendelian randomization; HEIDI, heterogeneity in dependent instruments.

In MR analysis, genetically proxied CCBs and vasodilator antihypertensives were identified to be associated with decreased risk of kidney cancer, while genetically proxied ACEIs were associated with increased risk of kidney cancer. Furthermore, genetically proxied antiadrenergic agents and centrally acting antihypertensives were found to be linked with decreased risk of ccRCC. We conducted SMR analysis to delve into whether genetically predicted levels of the targeted genes of these drugs were causally associated with kidney cancer. As shown in **Figure 4**, 4 targets presented causal associations with kidney cancer. As targets of CCBs, genetically predicted levels of *CACNA1C* (OR=4.64, 95% CI: 1.13-19.0, p=0.033) and *CALM1* (OR=1.37, 95% CI: 1.01-1.85, p=0.045) might increase the risk of kidney cancer. Genetically predicted levels of 2 targets of ACEIs presented different effect.* ACE* could decrease the risk of kidney cancer (OR=0.42, 95% CI: 0.18-0.99, p=0.046), while *LTA4H* could increase the risk of kidney cancer (OR=1.14, 95% CI: 1.05-1.24, p=0.002). As genetically proxied ACEIs were associated with increased risk of kidney cancer, the results of *LTA4H* should be interpreted with caution. HEIDI test indicated that these causal associations were not caused by LD. Overall, the association between the aforementioned drugs and the risk of kidney cancer might be associated with targets identified in SMR analysis.

### 3.4. Mediating roles of blood cells, blood lipids and anthropometric parameters in the associations between antihypertensive drugs and kidney cancer

**Figure 5:**
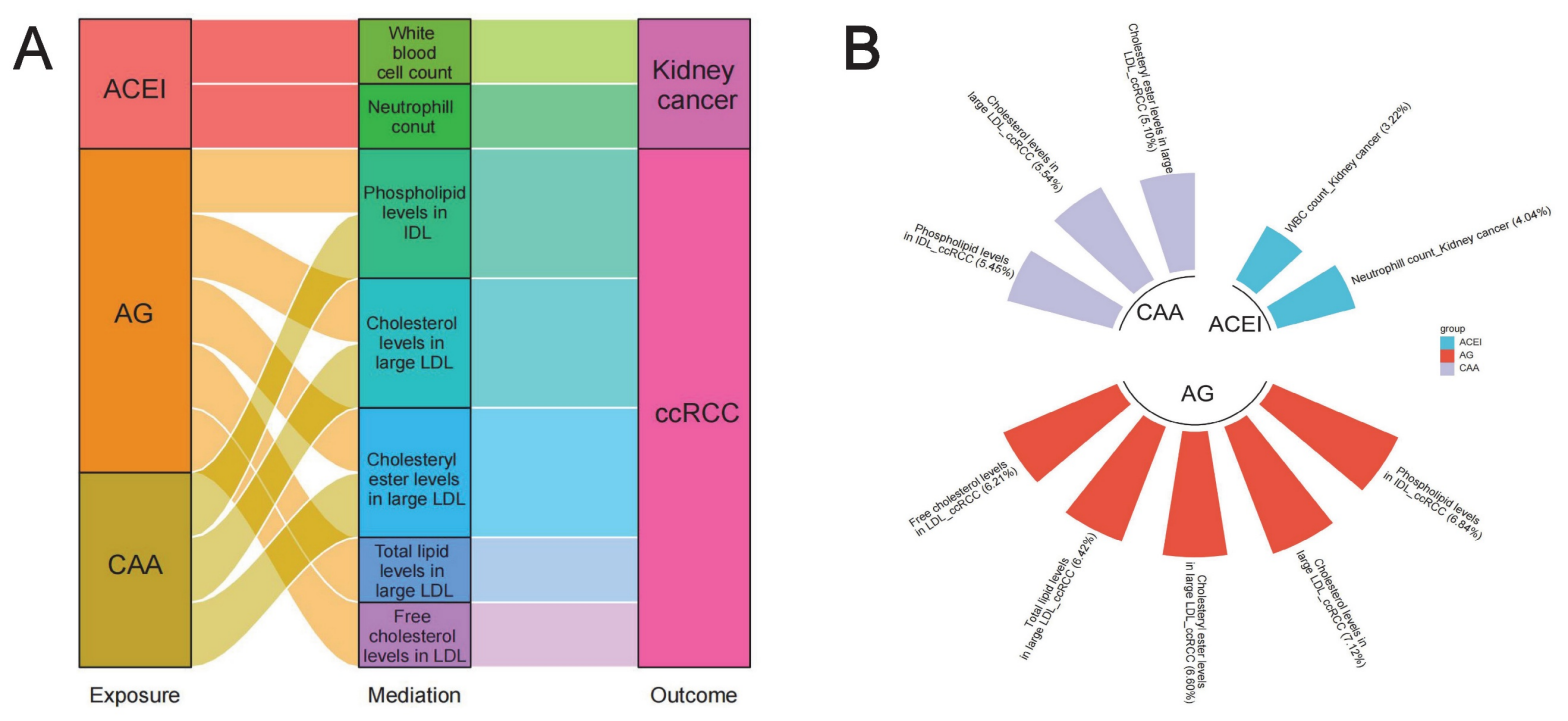
Two-step network mediation analysis connecting genetically proxies for antihypertensive drugs to kidney cancer through potential mediators. (A) The overview of pathways linking antihypertensive drugs to kidney cancer; (B) The proportion of association between genetically proxies for antihypertensive drugs and kidney cancer mediated by potential mediators. The bar chart is labeled as "mediator_ outcome (mediating proportion)". Abbreviations: ACEI, Angiotensin converting enzyme inhibitors; AG, Antiadrenergic agents; CAA, centrally acting antihypertensives; ccRCC, clear cell renal cell carcinoma; HDL, High density lipoprotein; IDL, Intermediate density lipoprotein; LDL, Low density lipoprotein.

As previous studies reported that antihypertensive drugs exerted specific regulatory effects on the levels of blood lipids and blood cells, and may also affect some anthropometric parameters (such as BMI) 37-41, we further investigated whether these variables played potential mediating roles in the associations between antihypertensive drugs and kidney cancer. Results of two-sample MR analyses are presented in **Table S11** and **S14**, and all have passed Steiger filtering test (**Table S18**). Although heterogeneity was detected in some associations (**Table S12** and **S15**), limited indication of horizontal pleiotropy was observed (**Table S13** and **S16**). Furthermore, two-sample network MR analyses were conducted to construct a mediating network connecting antihypertensive drugs and kidney cancer through potential mediators. Finally, a total of 10 significant (*p* < 0.05) pathways with directional consistency were identified (**Figure 5A**). The mediation proportion ranged from 3.22% to 7.12% (**Figure 4B**). Detailed results of mediation analysis are presented in **Table S17**. In summary, ACEIs elevated the risk of kidney cancer by increasing the levels of white blood cells and neutrophils, while antiadrenergic agents and centrally acting antihypertensives mitigated the risk of ccRCC by decreasing blood lipid levels.

### 3.5. Single-cell analysis revealed the expression patterns of four identified antihypertensive drug targets

**Figure 6:**
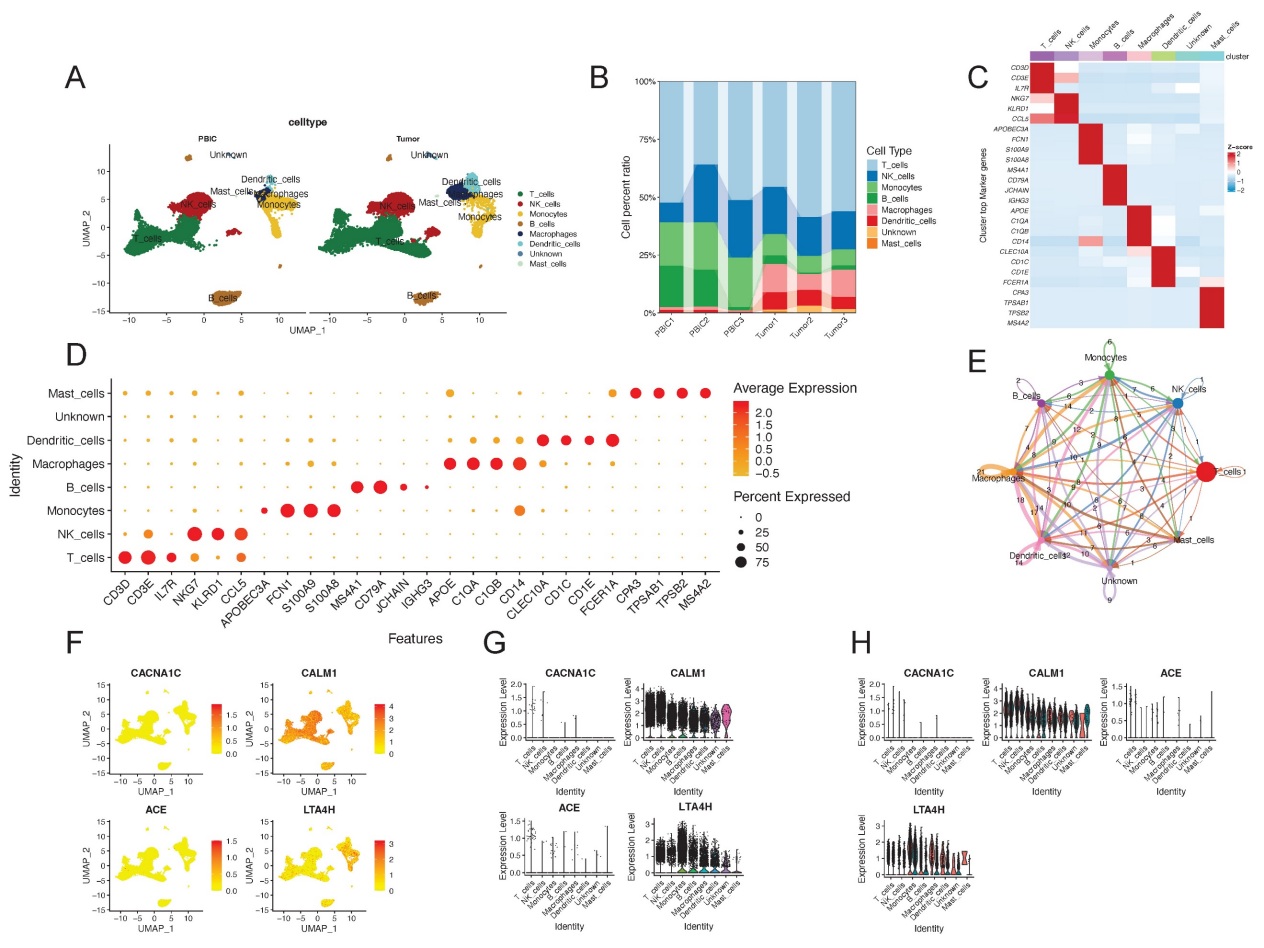
Single-cell analysis revealed the expression patterns of four identified antihypertensive drug targets causally associated with kidney cancer. (A) UMAP plot of the identified cell clusters in PBIC and tumor from renal cell carcinoma patients. (B) The composition of each cell type. (C) Heatmap distribution of marker genes in each cell type. (D) Bubble plot of the average and percent expression of marker genes in each cell type. (E) Cell-cell communications among cell types by Cellchat analysis. (F) and (G) show the expression patterns of the four identified antihypertensive drug targets in each cell cluster. (H) Violin plots of the expression of the four identified targets in different cell types and tissue types. Red represents peripheral blood immune cells and cyan represents tumor-infiltrating immune cells. Abbreviations: UMAP, Uniform manifold approximation and projection.

To further investigate the expression patterns of identified targets, we obtained scRNA-seq data and conducted single-cell analysis. We identified a total of 24,698 immune cells in 3 peripheral blood samples and 3 tumor samples from RCC patients. These cells were categorized into 7 different types, including T cells, NK cells, monocytes, B cells, macrophages, dendritic cells and mast cells (**Figure 6A**). The cell proportions in each sample are showed in **Figure 6B**. The annotated cell types were confirmed through the expression of some marker genes, which were presented in a heatmap and a bubble plot (**Figure 6C** and **6D**). We also found that different cell types interacted in diverse and distinct manners by Cellchat analysis (**Figure 6E**). In **Figure 6F-H**, we could find that *CALM1* and *LTA4H* widely expressed in different cell types in both peripheral blood and tumor tissue. *CALM1* exhibited high expression in NK cells and T cells, while *LTA4H* was highly expressed in monocytes. No significant difference in their expression levels was observed between peripheral blood and tumor tissue. Overall, single-cell analysis revealed the expression patterns of the four identified targets in different cell types and tissue types.

## 4. Discussion

### 4.1. Summary of the MR study

Previous studies have yielded somewhat conflicting results regarding the potential carcinogenesis of antihypertensive drugs 63-67. The inconsistent results may be attributed to diverse study designs, different races and sample sizes, distinctive comparators, varied durations of follow-up, the polygenic and multifactorial characteristics of kidney cancer with complex traits, and residual confounders caused by unmeasured factors 68. Therefore, the evidence level of previous studies is relatively low, which are unable to accurately establish a causal relationship between antihypertensive drugs and kidney cancer. MR studies are becoming increasingly popular in genetic epidemiology to draw definitive conclusions regarding the causality of association between exposures and outcomes by considering genetic variants as instrumental variables. The conclusions drawn from MR analysis are robust and of high quality, often rivaling those obtained from RCTs in terms of evidence strength. 69. In this MR study using genetic variants obtained from large-scale summary statistics, the association between genetic proxies for antihypertensives and the risk of kidney cancer was observed through meta-analysis. In the main analysis, genetically proxied CCB and vasodilator antihypertensives were suggestively related to decreased risk of kidney cancer, while genetically proxied ACEI was associated with increased risk of kidney cancer. Regarding specific RCC histological type, genetically proxied antiadrenergic agents and centrally acting antihypertensives were suggestively associated with a decreased risk of ccRCC, whereas there was a null association between pRCC or chRCC and genetic proxies for all 12 classes of antihypertensive drugs. Furthermore, previous studies attributed the effect of antihypertensive drugs on cancer risk solely to blood pressure modulation, and the specific mechanisms remain unclear. In our study, new insights were provided by integrating various novel analytical strategies. Specifically, we applied SMR, two-step network MR, and single-cell analysis. Summary-data-based MR analysis revealed that these suggestively significant associations might be linked to *CACNA1C*, *CALM1*, *ACE*, and *LTA4H*. Two-sample network MR analyses identify 10 significant (*p*<0.05) pathways linking antihypertensive drugs and kidney cancer risk. We found that ACEI elevated the risk of kidney cancer by increasing the levels of white blood cells and neutrophils, while antiadrenergic agents and centrally acting antihypertensives mitigated the risk of ccRCC by decreasing blood lipid levels. Single-cell analysis revealed the expression patterns of the four identified targets in different cell types and tissue types.

Using cis-acting variants in genes encoding antihypertensive drug targets as instrument proxies could avoid reverse-causality bias and minimize confounding by other determinants in a similar pattern as RCTs 69,70. The F-statistic of all selected SNPs genetically proxying for 12 antihypertensive drug classes was > 10, indicating that sufficient strength could ensure the validity of SNPs. The results of positive control analyses with hypertension and CAD ensure the plausibility and validity of genetic instruments of 12 classes of antihypertensive drugs. These genetic instruments facilitated the evaluation of the effect of typically decades-long use of antihypertensive medication, so the current study is more appropriate to access the directions of associations than to provide the magnitude of associations. Moreover, various sensitivity analyses with different assumptions were undertaken to interrogate the robustness of the main results. Agreement in the presence and direction of causal associations from multiple sensitivity analyses were observed, which enhanced the robustness and precision of causal estimates in the main analysis 45,46. Overall, the MR study based on the large-scale available GWAS and eQTL data sets could overcome some of the caveats of observational studies and RCTs, such as the limitation of sample size and feasibility, reverse causality, and residual confounders 22. The specific implications of our findings are that we have not only clarified the causal associations between antihypertensive drugs and kidney cancer risk but also preliminarily explored the underlying mechanisms and identified important target genes. These results may provide guidance for the development of new antihypertensive drugs and the optimization of treatment strategies for hypertension in patients with high kidney cancer risk.

### 4.2. Explanation of the MR results

MR analyses based on both the GWAS dataset and the eQTL dataset suggested that genetic proxies for ACEIs increase the risk of kidney cancer, which is consistent with some previous studies 71,72. The potential carcinogenic role of ACEIs has been reported in some different types of human cancers such as lung cancer, kidney cancer, and melanoma 72-76. Proto-oncogenes, oncogenes, cell signaling, microRNAs, and epigenetic factors in the renin-angiotensin system (RAS) are deemed to play important roles in cancer development 77,78. As a multifaceted enzyme, ACE is capable of cleaving several different peptide substrates with potential roles in carcinogenesis 79. Inhibition of ACE could lead to the accumulation of bradykinin and substance P, which potentially mediates tumor growth and proliferation 68,80. Previous studies demonstrated that ACEIs often encouraged invasive potential and vascular endothelial growth factor (VEGF) production, which in turn boost angiogenesis and pro-tumorigenic transcription factors. ACEIs could also promote inflammation and participate in metastasis invasion, and migration processes 74,78,81. However, some experimental and observational studies reported that the use of ACEIs confers a protective effect against cancer, which could suppress tumor growth, and inhibit tumor angiogenesis and metastasis 82-84. Therefore, the association between ACEIs and kidney cancer remains controversial and warrants further research to elucidate. Our study also found that ACEIs may increase the risk of kidney cancer by increasing the levels of white blood cells and neutrophils, but there is limited research on this topic. In the future, focusing on this field might provide more credible evidence to reveal the exact association between ACEIs and kidney cancer risk.

Different from ACEIs, CCBs and vasodilator antihypertensives were suggestively related to decreased risk of kidney cancer in the main analyses. A meta-analysis of five RCTs, including 5,451 CCBs users and 5,207 nonusers followed for several years, showed a lower risk for malignancy among CCBs users when compared to nonusers (OR: 0.78, 95% CI: 0.60-1.00), which is consistent with our results 85. Indeed, the association between CCBs and cancer has been reported to range from a protective effect to neutral risk 65,85-88, and some studies have shown that the use of CCBs increases the risk of cancer 68,73,89,90. The inconclusive findings from previous studies mirror the complex biological mechanisms associating CCBs with cancer. The mechanism behind the effect of CCBs on kidney cancer risk is unclear, but experimental data could provide some insights. Prior experimental studies showed that both low and high dose verapamil substantially enhanced tumor apoptosis, and reduced tumor cell growth and metastasis 91. The pro-apoptotic effects of verapamil may be explained by its actions as a CCB. Calcium ions (Ca^2+^) is toxic at high concentrations, thus verapamil may help to foster apoptosis through disruption of the Ca^2+^ balance 91. Prior in-vitro and animal studies also suggested that CCBs may regulate cell proliferation and calcium influx, thereby inhibiting the proliferation of calcium-dependent neoplastic cells 16. However, the experimental and clinical evidence on the association between CCBs and kidney cancer is few, and further investigations are needed. Regarding vasodilator antihypertensives, several drugs are endothelin receptor antagonists (ERAs), including bosentan, ambrisentan, and sitaxentan 92. The endothelin axis has pleiotropic functions associated with hypertensive pathologies and some fundamental cellular processes such as cell proliferation and apoptosis 92. The endothelin group comprises the three peptide isoforms, ET-1, ET-2, and ET-3, which have distinct tissue distributions 92. ET-1 is a mitogenic and antiapoptotic peptide 93. Preclinical animal experiments and cellular models have demonstrated that ET-1 could induce VEGF expression by increasing levels of hypoxia-inducible factor 1α (HIF-1α), which plays an important role in the development of kidney cancer 94. Elevated levels of HIF-1α are strongly correlated with angiogenesis, cancer resistance, metastasis, and poor prognosis 95. VEGF could stimulate cancer cells and fibroblasts to produce proangiogenic proteases resulting in tumor angiogenesis 96. Moreover, ET-1 directly and indirectly promotes the epithelial-mesenchymal transition (EMT), invasion, and metastasis of cancer cells 97. ERAs may be useful in cancer prevention and treatment by targeting endothelin. Several ERAs have exhibited promising effects of greatly reducing the proliferation and invasion of tumor cells in the context of experimental cancer 98,99. However, evidence of ERAs and the risk of kidney cancer is lacking. Clinical trials with ERAs in the treatment of different of different types of cancer have not been able to produce measurable statistically significant positive results 100,101. The lack of effective clinical evidence and the discrepancy between the results from preclinical models and the human clinical trials call for further exploration in the future.

When considering specific RCC histological types, our results showed that genetically proxied antiadrenergic agents and centrally acting antihypertensives were suggestively associated with a decreased risk of ccRCC. For pRCC and chRCC, we found a null association between them and genetic proxies for all 12 classes of antihypertensive drugs. Emerging molecular and epidemiological evidence suggests that antiadrenergic agents may have both preventive and direct therapeutic actions in the treatment of kidney cancer. For example, as an important member of antiadrenergic agents, α1-adrenoreceptor antagonists are currently used in the treatment of hypertension, renal and ureteric stones, as well as benign prostatic hyperplasia (BPH) 102. Evidence at the cellular level suggested that the antitumor effect of α1-adrenoreceptor antagonists in kidney cancer proceeds via reducing vascularity and impairing growth within the tumor microenvironment 102. Some α1-adrenoreceptor antagonists including Doxazosin and DZ-50, were both found to exert potent antitumor action against human RCC cell lines 786-O and Caki cells 103-106. DZ-50 had the chemoprotective potential to suppress angiogenesis and reverse the hypoxic nature of cancer by disrupting the tumor environment 107. Doxazosin and naftodipil, selective α1-adrenoceptor antagonists, were found to inhibit the proliferation of RCC cells both in vitro and in vivo human tumor xenografts in mice 104,106. However, only a few clinical studies have examined the relationship between antiadrenergic agents and cancer risk, and the results remain inconsistent 108. As for centrally acting antihypertensives, the evidence on the association between it and cancer is lacking, and the mechanism is still unclear. Our study found that centrally acting antihypertensives could reduce the levels of several types of blood lipids, and this might be the potential mechanisms. Further investigations are needed to validate our results.

### 4.3. Limitations

There are several limitations to this MR study. First, although we provided evidence of the association between genetic proxied antihypertensive drugs and kidney cancer, the estimates of the drug effects could not be explained as clinical effects of drugs. The size of the estimate should be interpreted cautiously because genetic variants reflect the effect of lifelong antihypertensive medications on the risk of kidney cancer, which might suggest larger risk reductions per unit change in antihypertensive drug targets compared with those obtained from drug administration over a relatively shorter duration. The true magnitude of risk changes through taking antihypertensive drugs might not correspond to the effect size observed in our study 69. Second, variables including drug dose, duration of exposure, interindividual variation in drug metabolism, ability to reach the tissue of relevance, and drug binding affinity play roles in modifying drug efficacy and toxicity, making it hard to extrapolate the actual effect of antihypertensive drug exposure from genetic analyses 22. Third, conventional MR analyses assume a linear relationship between genetically proxied exposures and outcomes, but some drugs may not trigger any biological response until a drug dose exceeds a certain level 23. Fourth, MR analyses are restricted to examining on-target effects of therapeutic interventions, so we could not evaluate the influence of off-target effects of antihypertensive drugs on the risk of kidney cancer. In addition, limited by GWAS summary statistics, we could not perform corresponding subgroup analyses and statistically compare the specific risk rates between the cases and controls, such as age, and gender. We did not investigate the genetically proxied association between drug target inhibition and other stratified kidney cancer phenotypes such as stage, grade, cancer aggressiveness, metastasis, prognosis, and recurrence, either. Available subgroup and stratified GWAS data would enable future MR analyses to investigate the influence of antihypertensive medications on the risk of kidney cancer in more detail. Moreover, combinations of antihypertensive drugs are usually used to treat hypertension in clinical practice. However, we could not assess the association between combinations of antihypertensive drugs and the risk of kidney cancer due to the design of the study being summary-data MR. Future exploration on this topic could focus on the effect of joint antihypertensive drugs based on individual-level data 64,109. Last but not least, the MR analyses were based on GWAS summary data of Europeans, which may not apply to other populations such as Asians. Further exploration of other ancestries would be worthwhile in the future.

## 5. Conclusions

Antihypertensive drugs represent one of the most commonly and frequently prescribed classes of drugs worldwide. Thus, rigorous monitoring and detection of adverse effects is recommended since even modest effects could have dramatic consequences on a large scale 110. Our MR analyses provide human genetic support for the safety profile of the antihypertensive drugs. Our findings demonstrated that genetically proxied long-term use of CCBs and vasodilator antihypertensives with a decreased risk of kidney cancer. We also provide evidence for a protective association of genetically proxied antiadrenergic agents and centrally acting antihypertensives with lower ccRCC risk. In contrast, genetically proxied ACEIs was related to an increased risk of kidney cancer, but it's still not sufficient to issue restrictive warning on the use of ACEIs. Further evidence from experimental studies and well-designed clinical trials is needed to confirm these findings.

## Supplementary Material

Supplementary tables.

## Abbreviations

ACEIs: angiotensin-converting enzyme inhibitors
ARBs: angiotensin II receptor blockers
BBs: beta-adrenoceptor blockers
BPH: Benign prostatic hyperplasia
Ca^2+^: Calcium ions
chRCC: Chromophobe renal cell carcinoma
Cis-eQTL: cis-expression quantitative trait loci
CI: confidence interval
ccRCC: Clear cell renal cell carcinoma
CAD: coronary artery disease
EMT: Epithelial-mesenchymal transition
ERA: Endothelin receptor antagonist
FDR: False discovery rate
GWASs: genome-wide association studies
HEIDI: Heterogeneity in dependent instruments
HIF-1α: Hypoxia-inducible factor-1α
IVs: Instrumental variables
IVW: Inverse-variance weighted
MR: Mendelian randomization
MAF: Minor allele frequency
MR-PRESSO: MR-pleiotropy residual sum and outlier
SMR: Summary-data-based mendelian randomization
SNP: Single nucleotide polymorphisms
OR: Odds ratio
pRCC: Papillary renal cell carcinoma
PSDs: potassium-sparing diuretics
PCA: Principal component analysis
RAS: Renin-angiotensin system
RCTs: randomized clinical trials
RCC: Renal cell carcinoma
RR: Relative risk
STROBE-MR: Studies in epidemiology using mendelian randomization
SE: standard errors
SBP: Systolic blood pressure
VEGF: Vascular endothelial growth factor
UMAP: Uniform manifold approximation and projection
